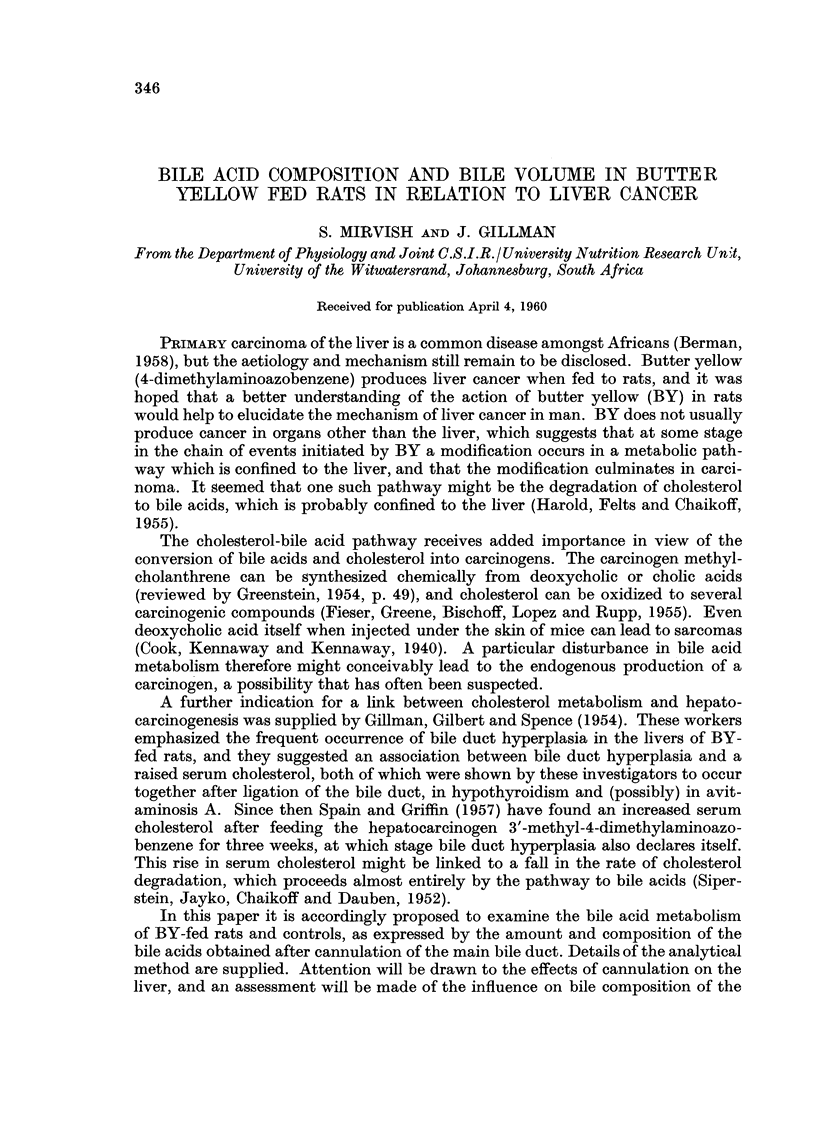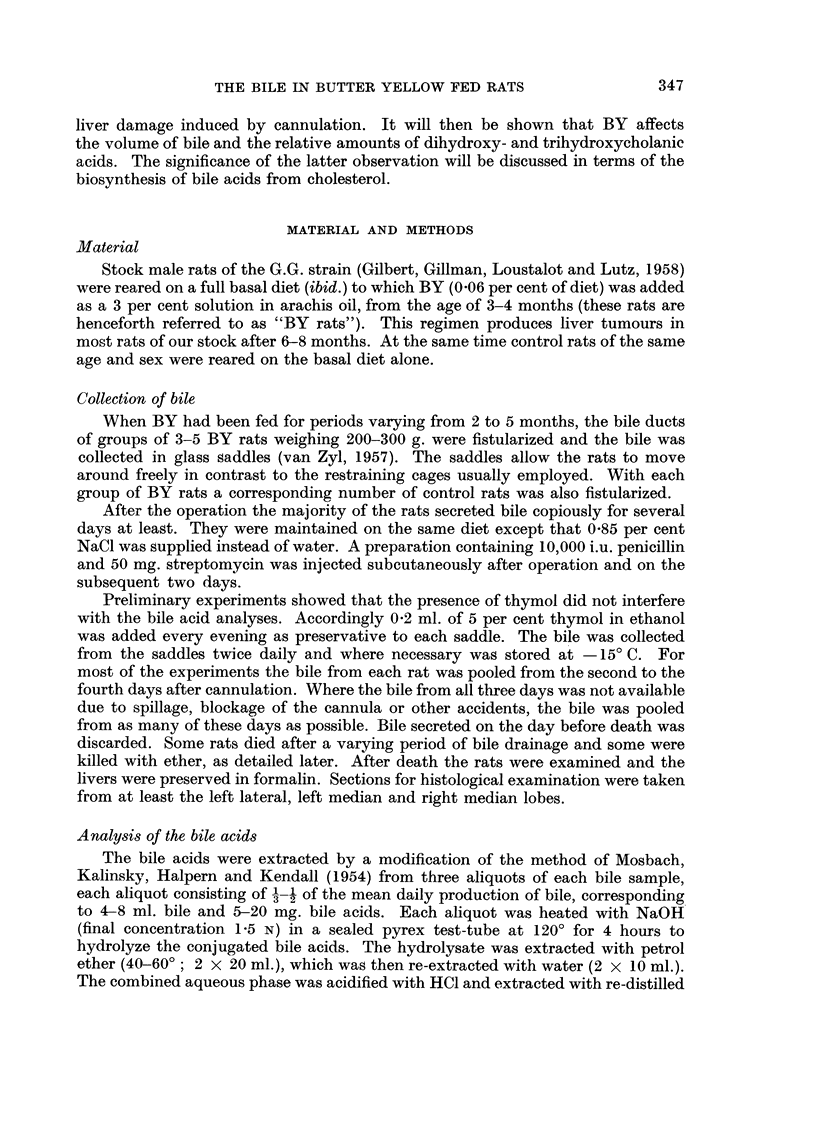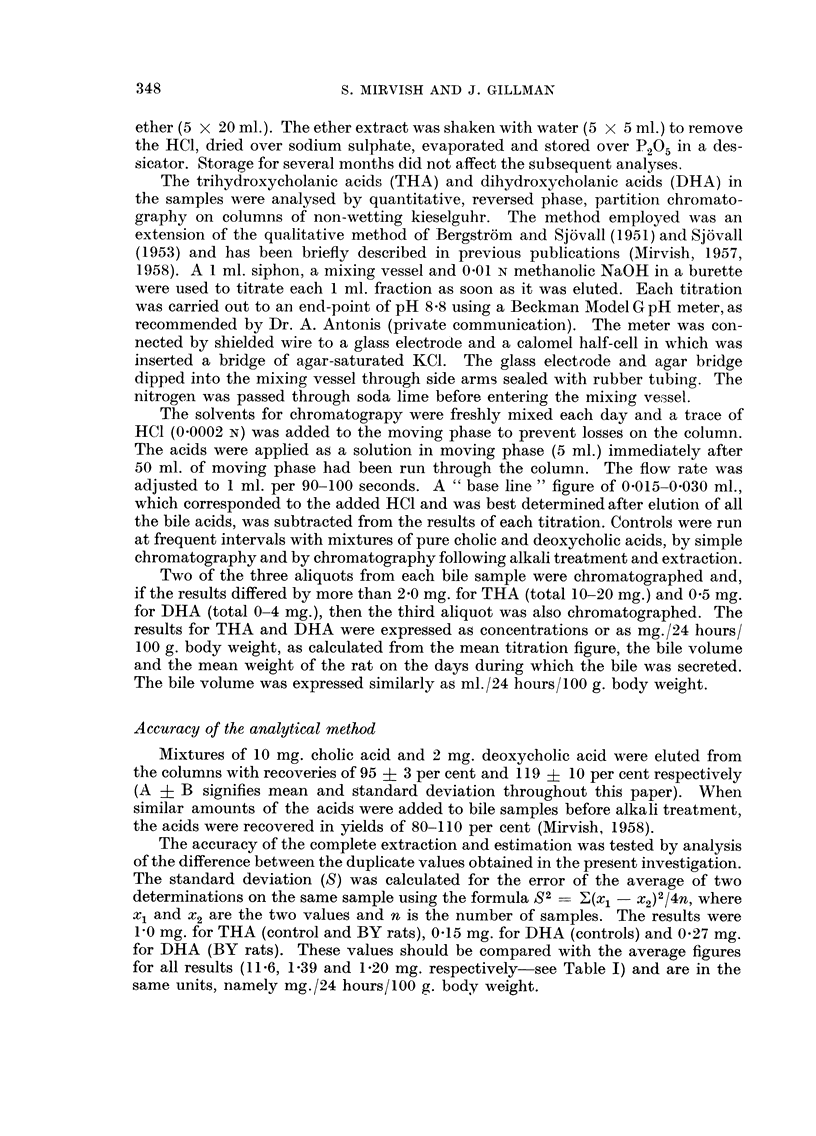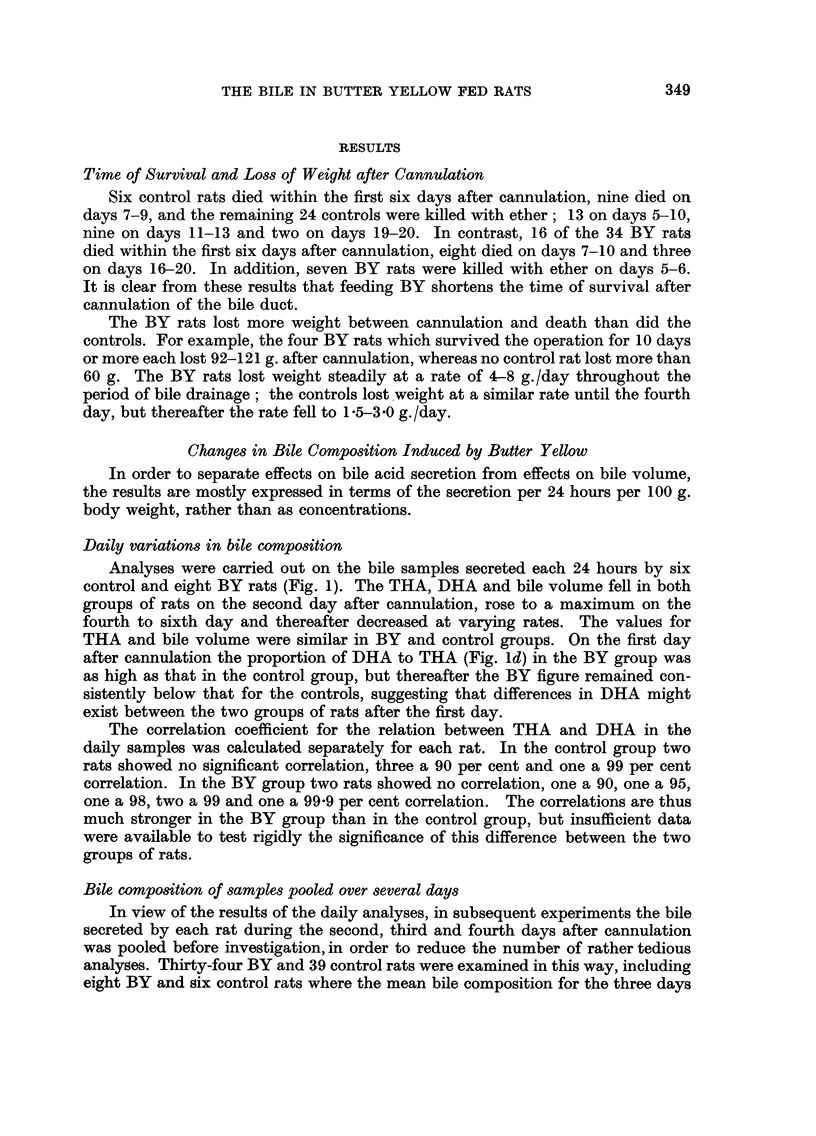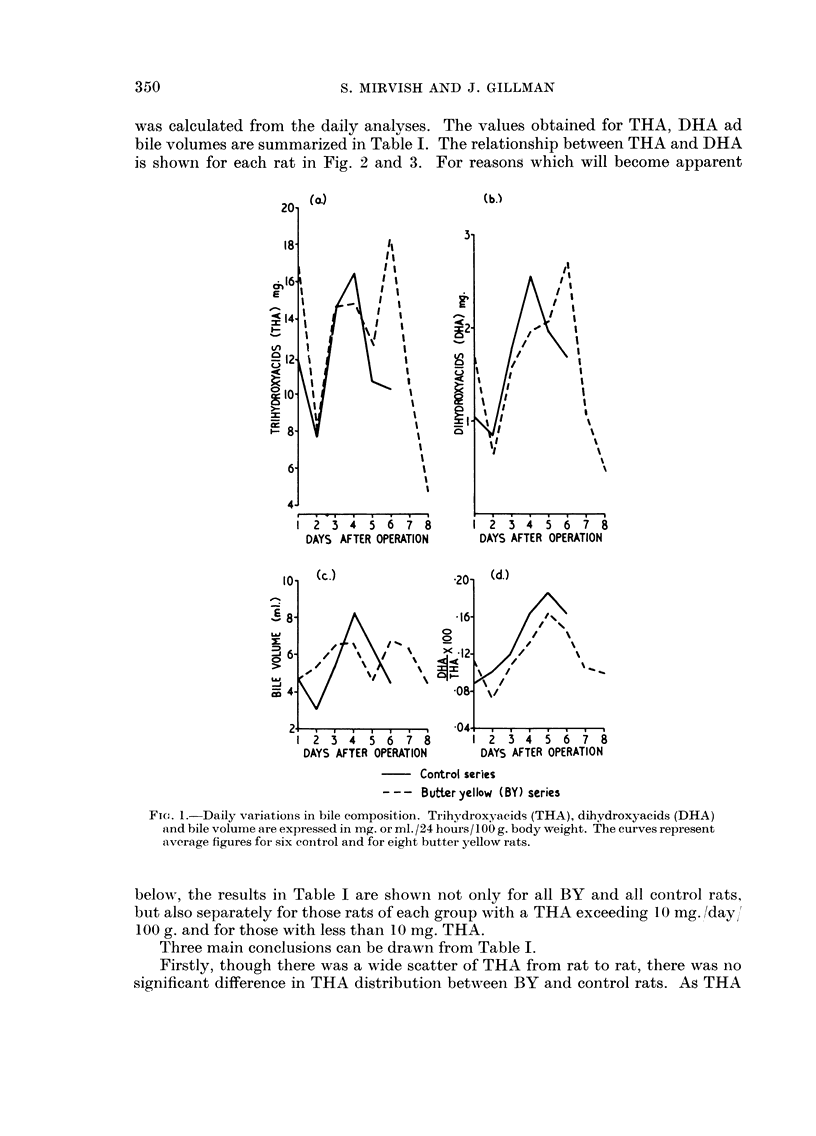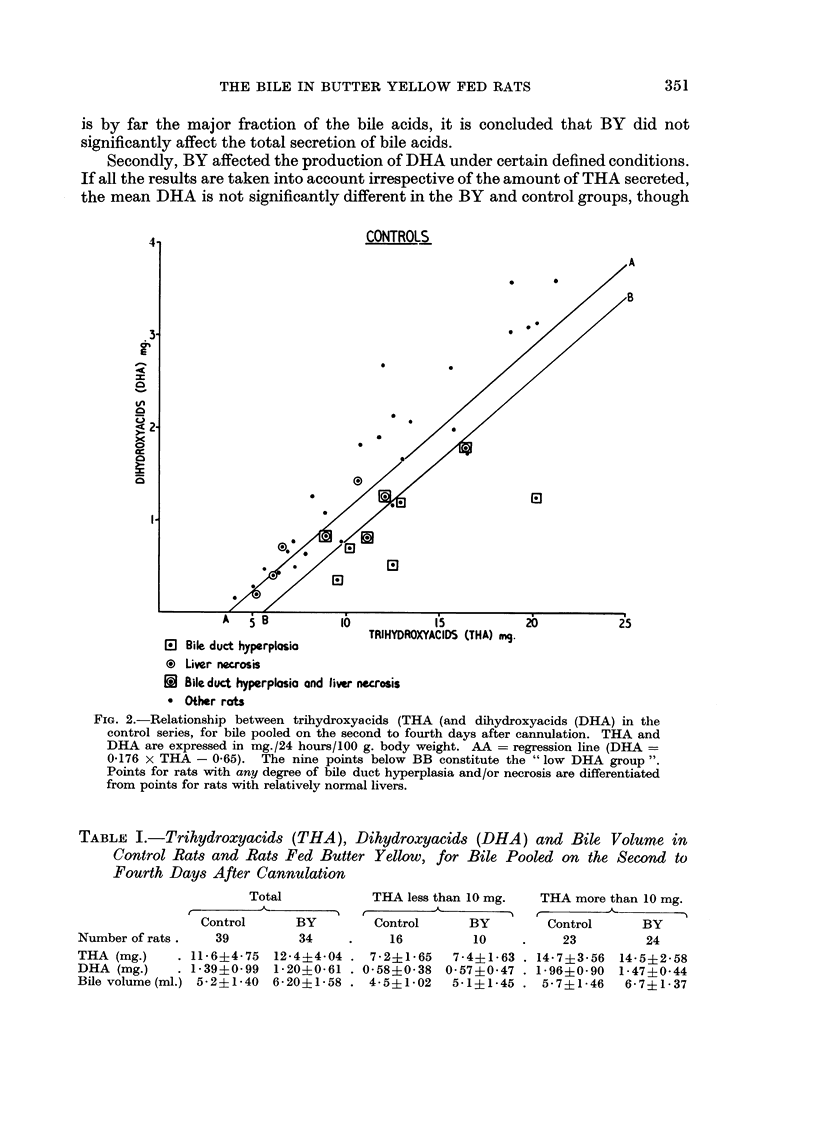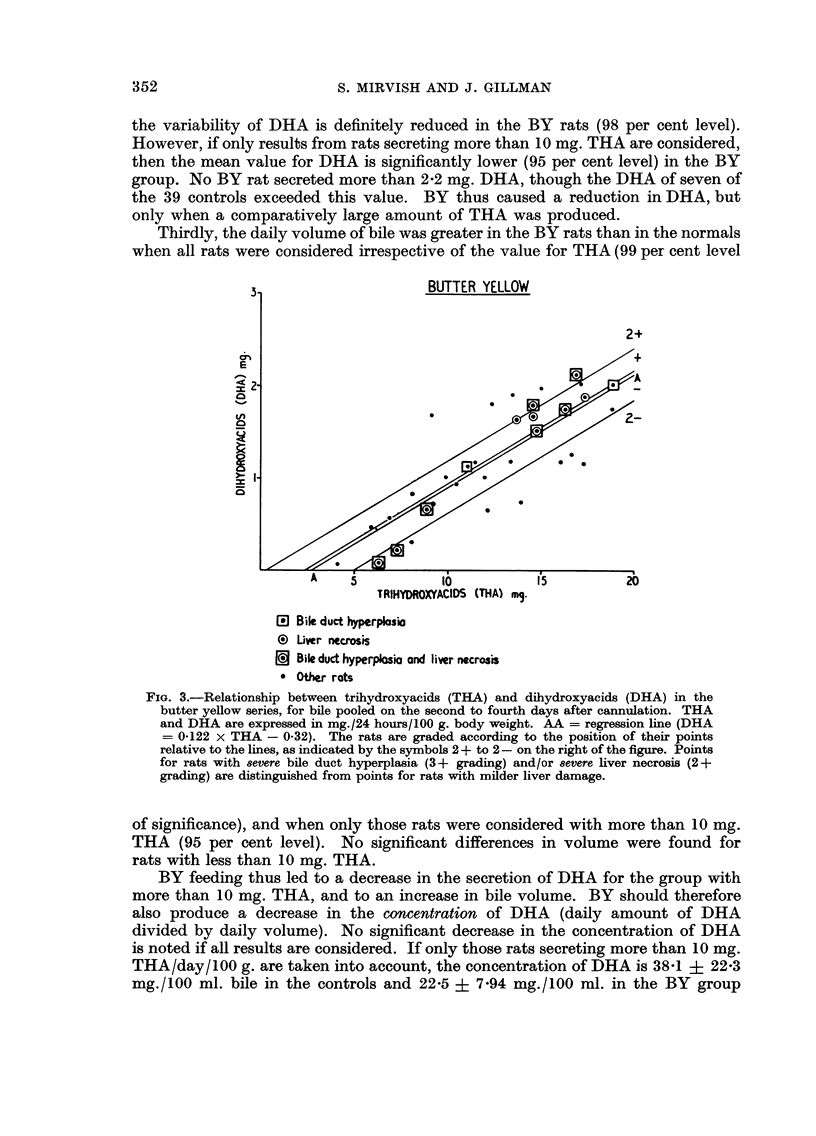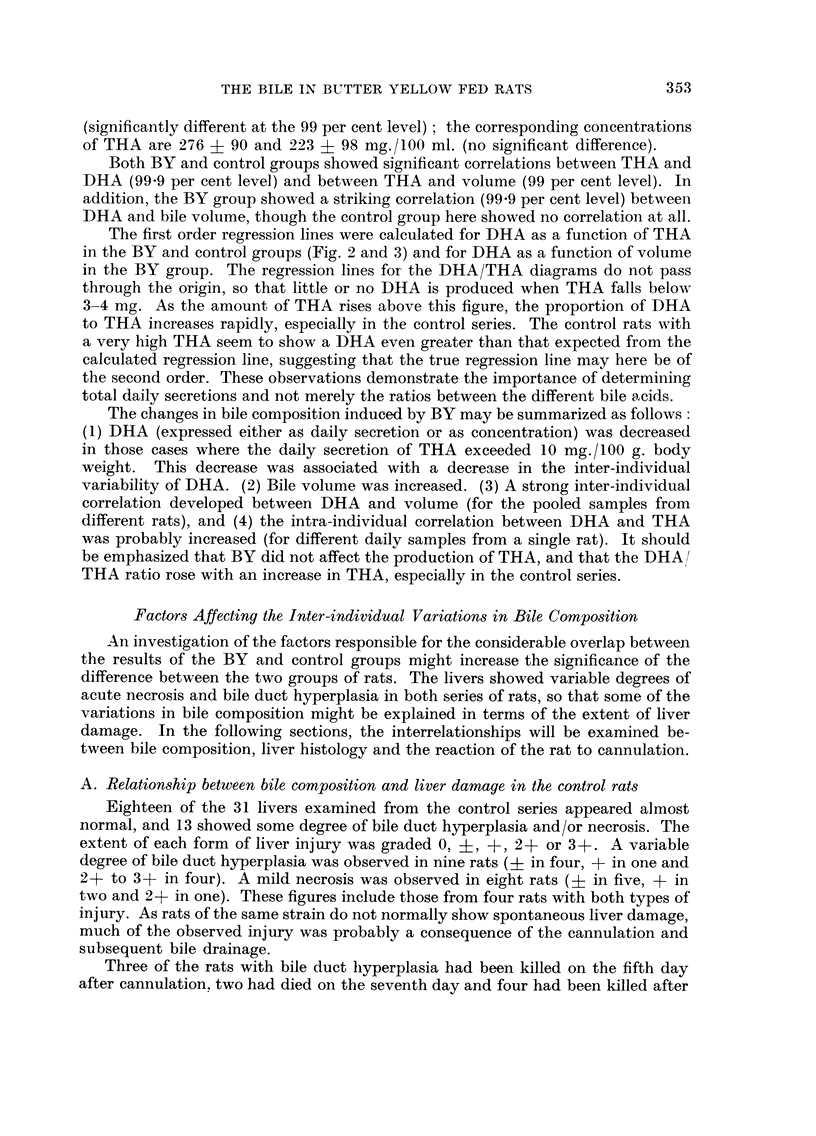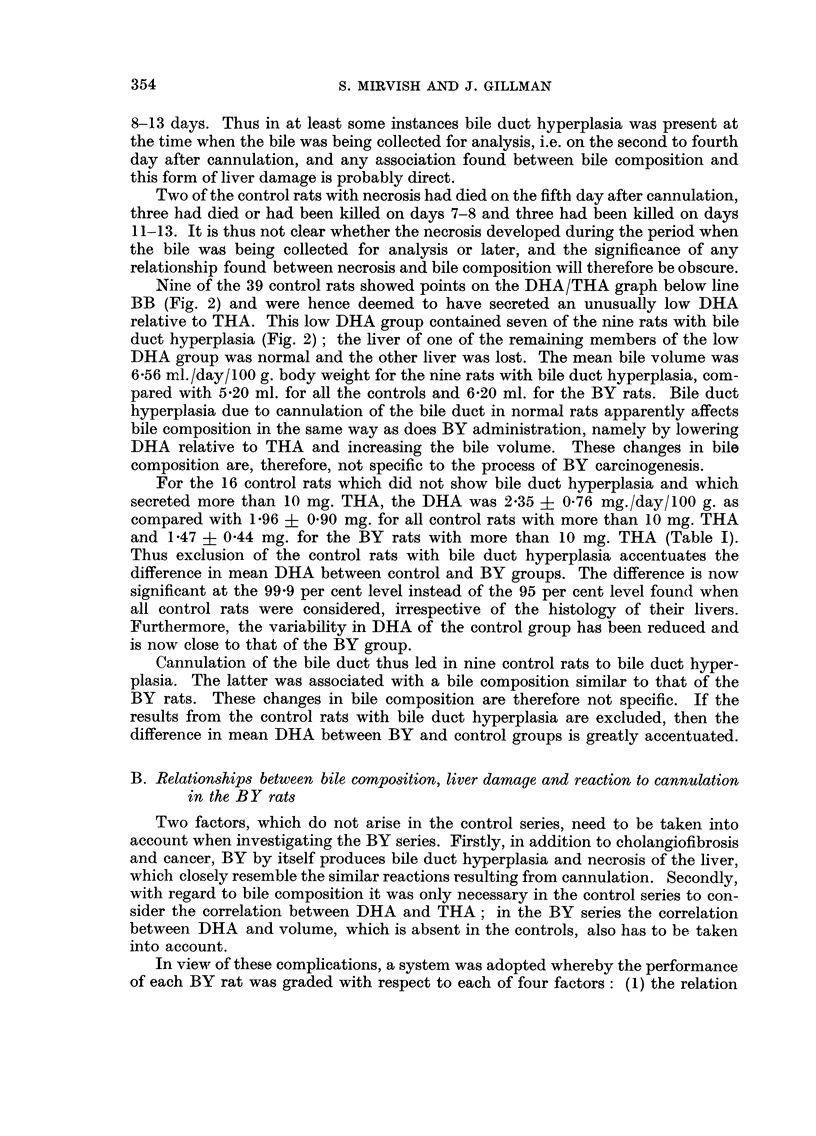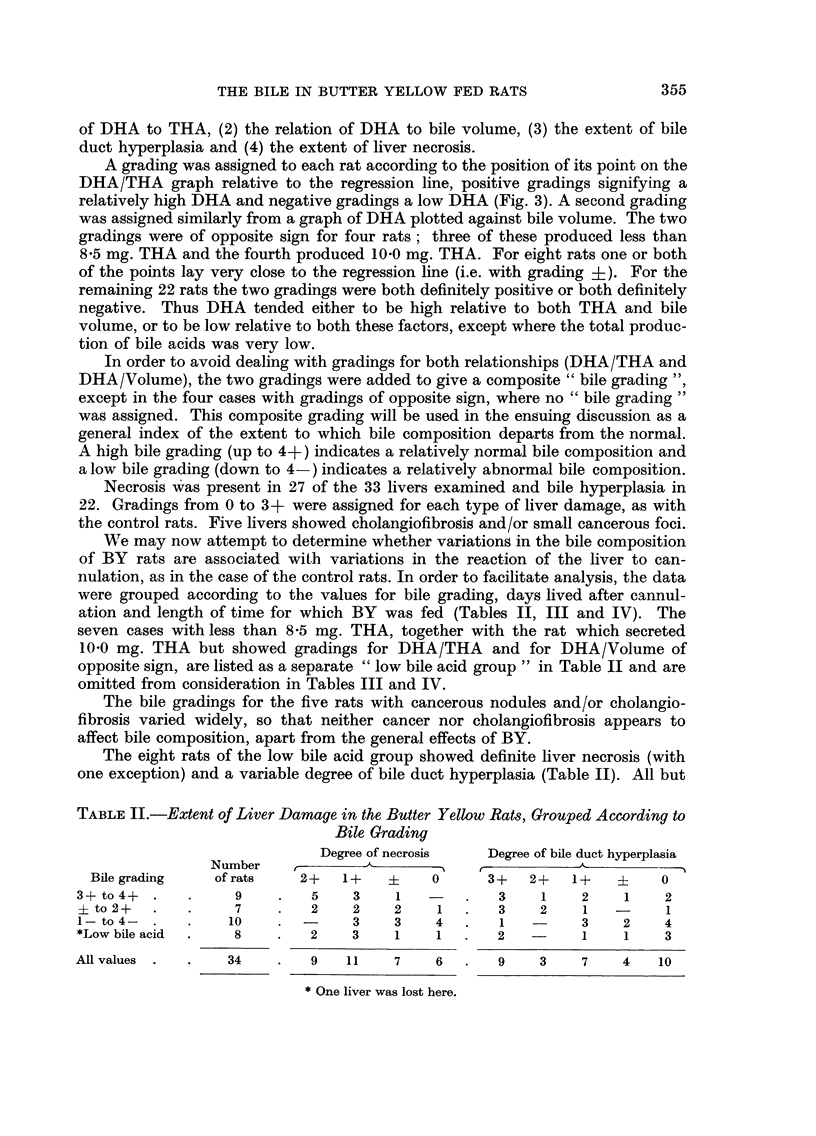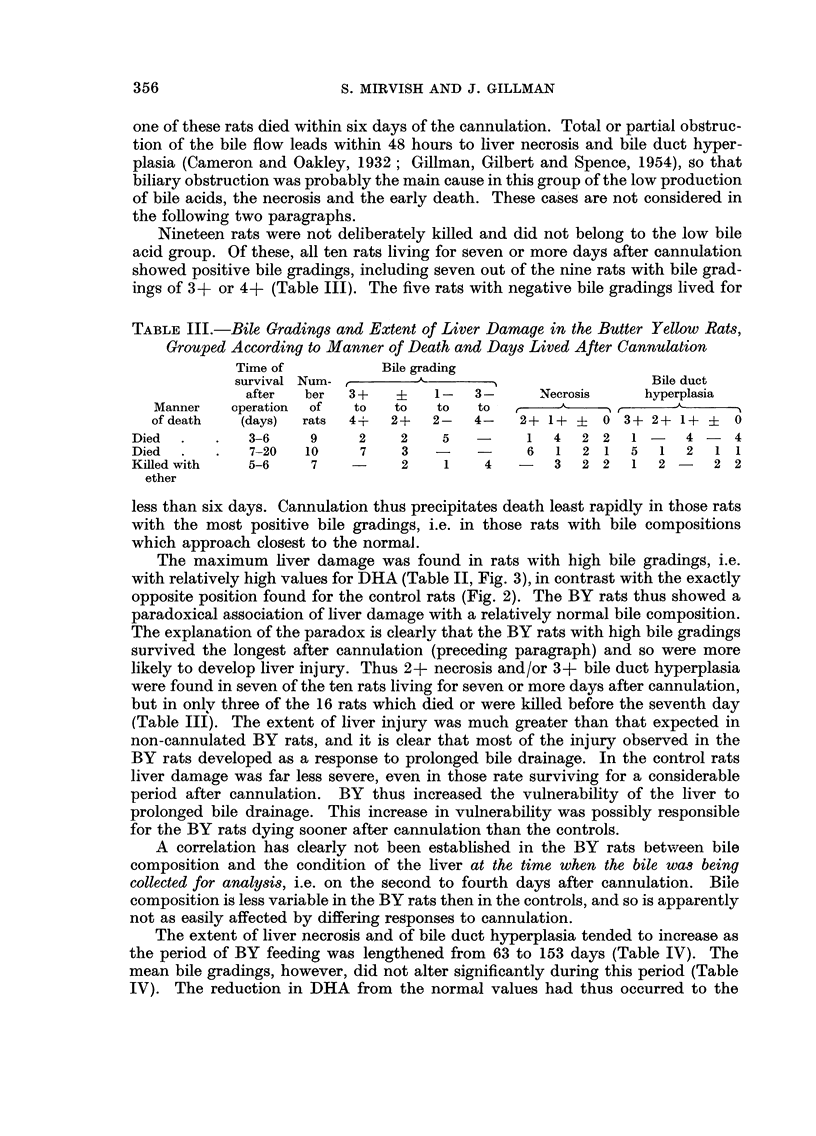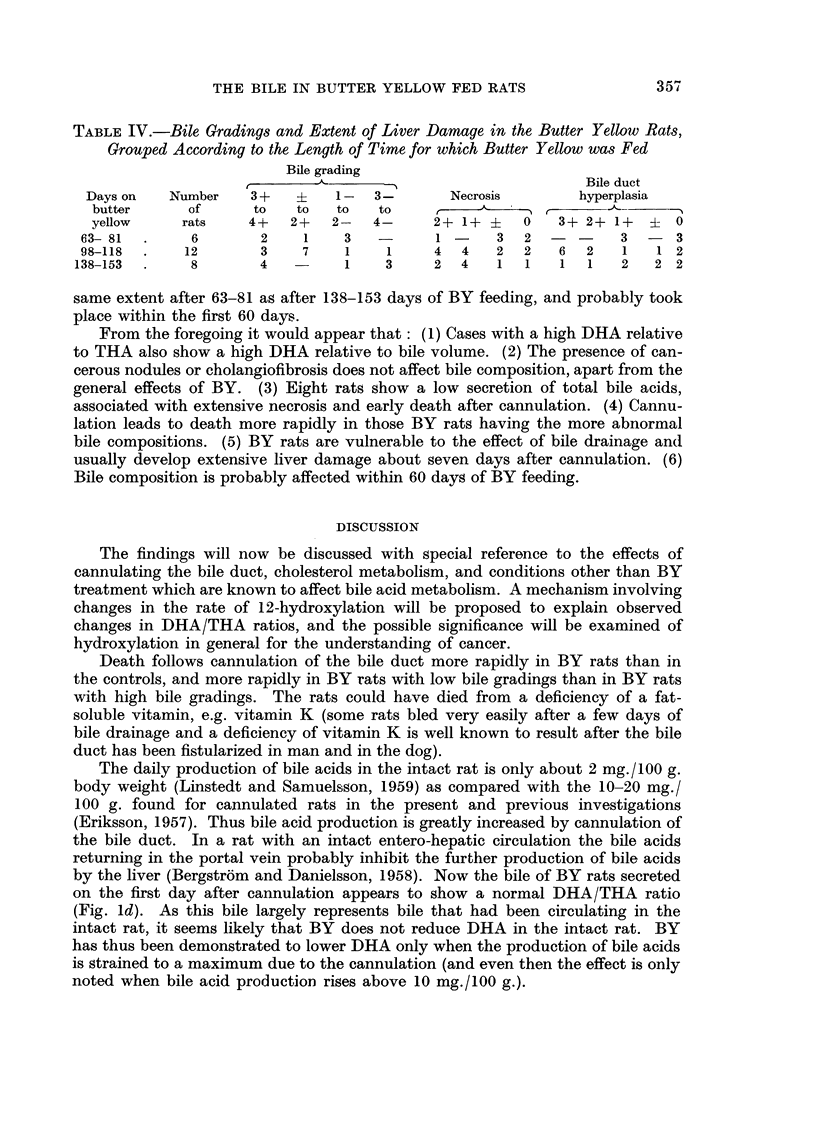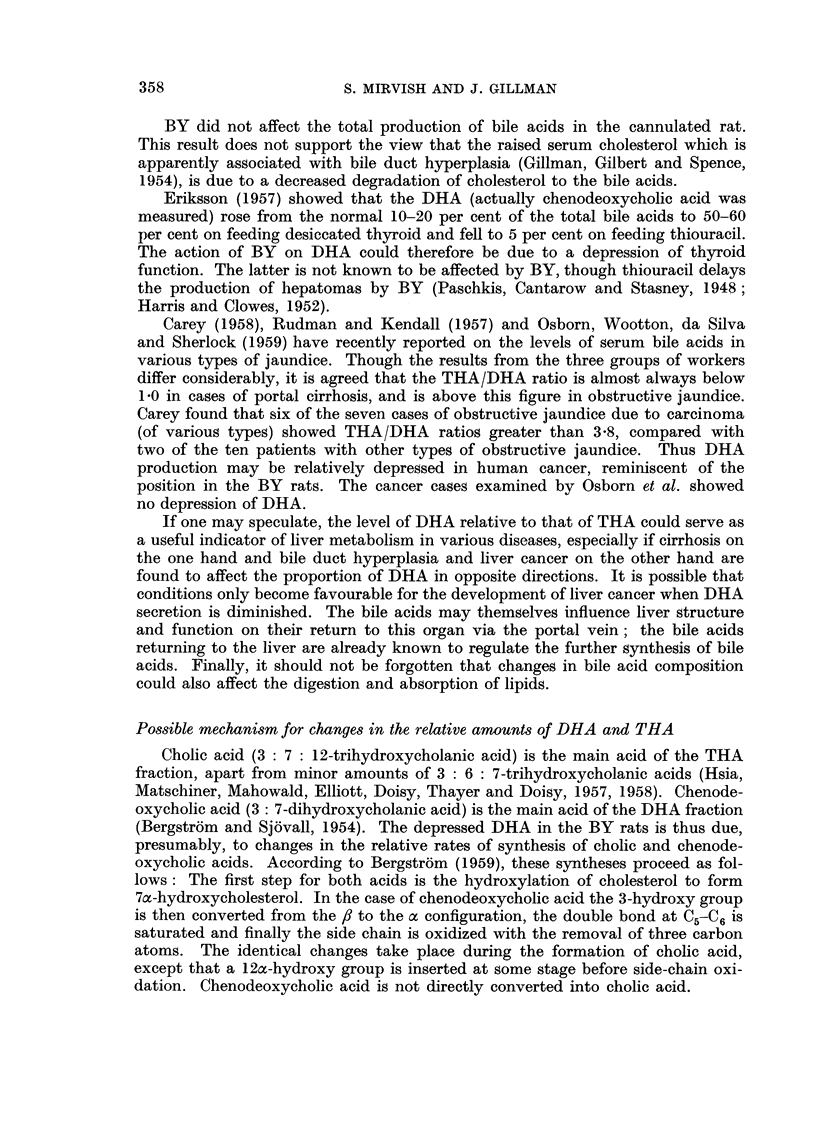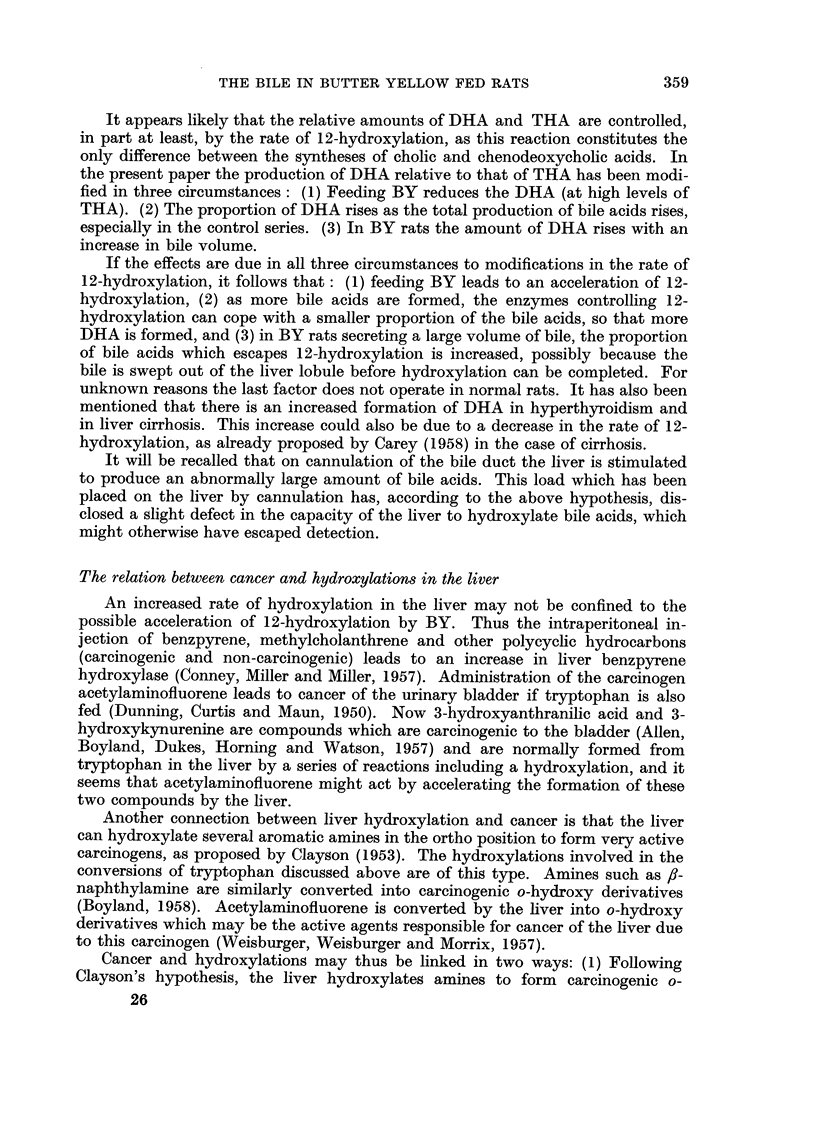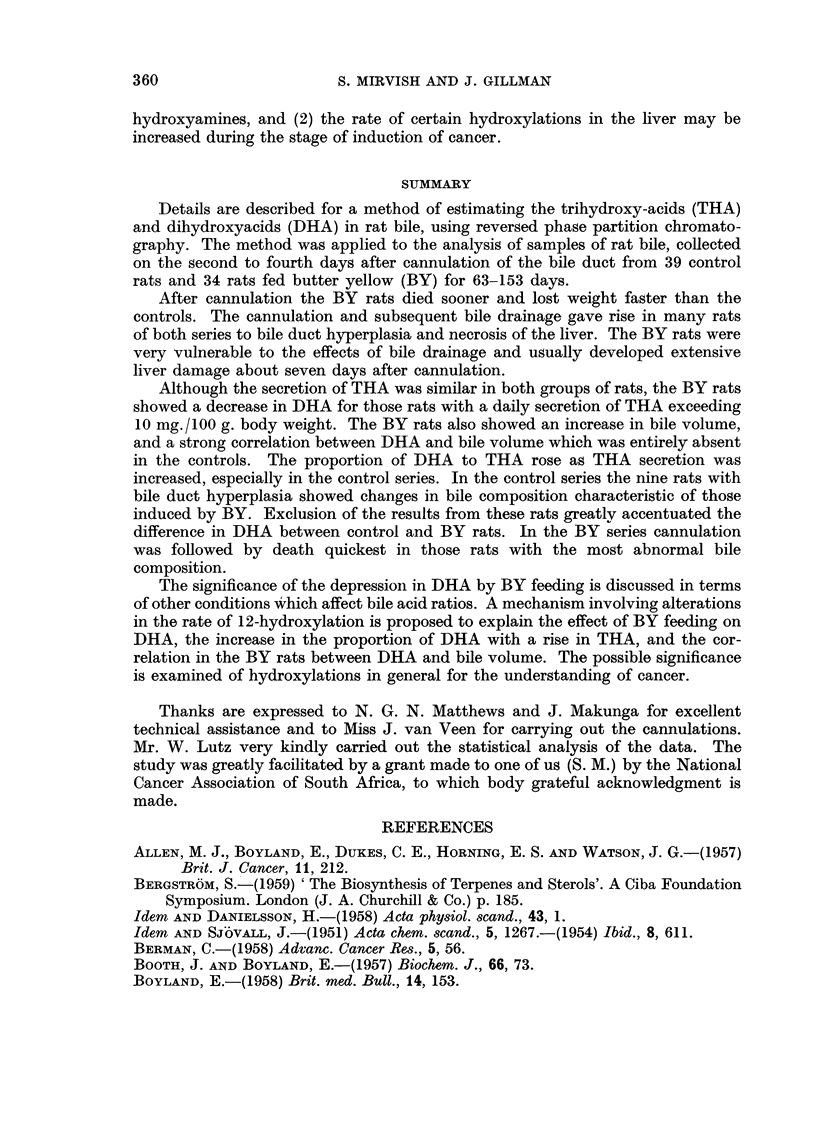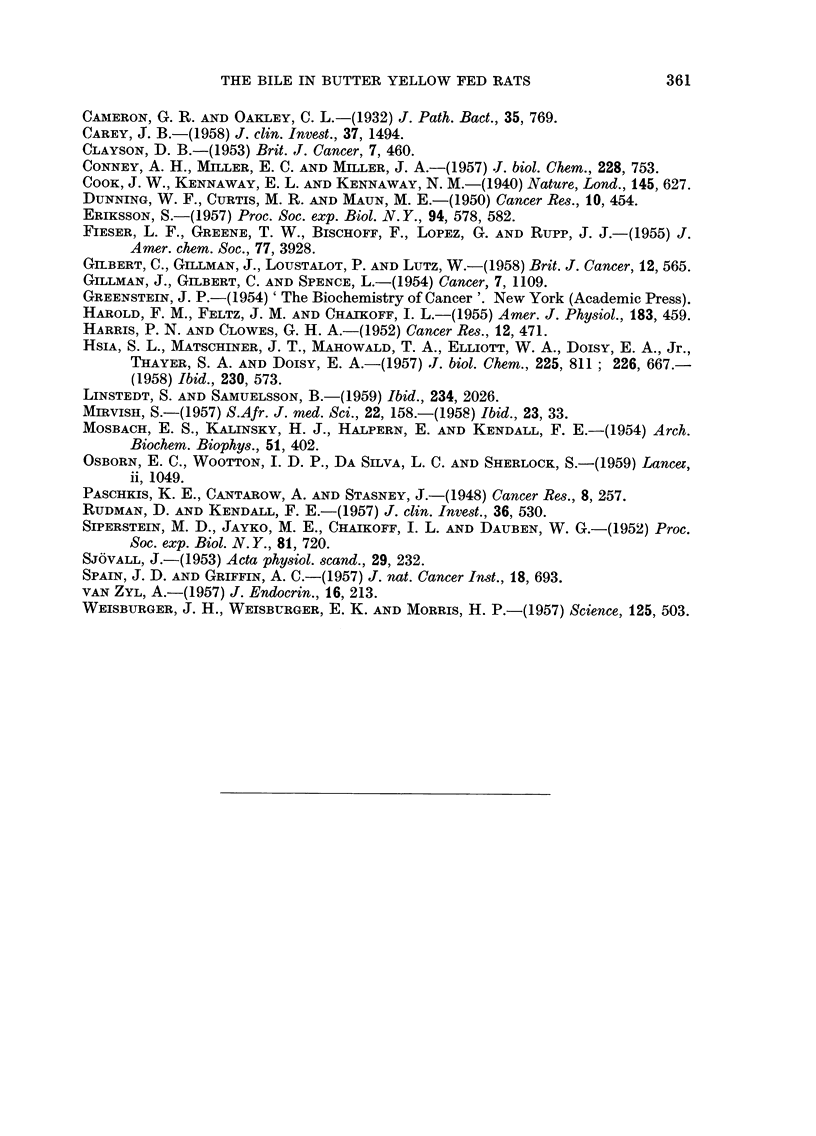# Bile Acid Composition and Bile Volume in Butter Yellow Fed Rats in Relation to Liver Cancer

**DOI:** 10.1038/bjc.1960.41

**Published:** 1960-06

**Authors:** S. Mirvish, J. Gillman


### BILE ACID COMPOSITION AND BILE VOLUME IN BUTTER YELLOW FED RATS IN RELATION TO LIVER CANCER

S. MIRVISH AND J. GILLMAN

*From the Department of Physiology and Joint C.S.I.R./University Nutrition Research Unit, University of the Witwatersrand, Johannesburg, South Africa*

Received for publication April 4, 1960

PRIMARY carcinoma of the liver is a common disease amongst Africans (Berman, 1958), but the aetiology and mechanism still remain to be disclosed. Butter yellow (4-dimethylaminoazobenzene) produces liver cancer when fed to rats, and it was hoped that a better understanding of the action of butter yellow (BY) in rats would help to elucidate the mechanism of liver cancer in man. BY does not usually produce cancer in organs other than the liver, which suggests that at some stage in the chain of events initiated by BY a modification occurs in a metabolic pathway which is confined to the liver, and that the modification culminates in carcinoma. It seemed that one such pathway might be the degradation of cholesterol to bile acids, which is probably confined to the liver (Harold, Felts and Chaikoff, 1955).

The cholesterol-bile acid pathway receives added importance in view of the conversion of bile acids and cholesterol into carcinogens. The carcinogen methylcholanthrene can be synthesized chemically from deoxycholic or cholic acids (reviewed by Greenstein, 1954, p. 49), and cholesterol can be oxidized to several carcinogenic compounds (Fieser, Greene, Bischoff, Lopez and Rupp, 1955). Even deoxycholic acid itself when injected under the skin of mice can lead to sarcomas (Cook, Kennaway and Kennaway, 1940). A particular disturbance in bile acid metabolism therefore might conceivably lead to the endogenous production of a carcinogen, a possibility that has often been suspected.

A further indication for a link between cholesterol metabolism and hepatocarcinogenesis was supplied by Gillman, Gilbert and Spence (1954). These workers emphasized the frequent occurrence of bile duct hyperplasia in the livers of BY-fed rats, and they suggested an association between bile duct hyperplasia and a raised serum cholesterol, both of which were shown by these investigators to occur together after ligation of the bile duct, in hypothyroidism and (possibly) in avitaminosis A. Since then Spain and Griffin (1957) have found an increased serum cholesterol after feeding the hepatocarcinogen 3'-methyl-4-dimethylaminoazobenzene for three weeks, at which stage bile duct hyperplasia also declares itself. This rise in serum cholesterol might be linked to a fall in the rate of cholesterol degradation, which proceeds almost entirely by the pathway to bile acids (Siperstein, Jayko, Chaikoff and Dauben, 1952).

In this paper it is accordingly proposed to examine the bile acid metabolism of BY-fed rats and controls, as expressed by the amount and composition of the bile acids obtained after cannulation of the main bile duct. Details of the analytical method are supplied. Attention will be drawn to the effects of cannulation on the liver, and an assessment will be made of the influence on bile composition of the liver damage induced by cannulation. It will then be shown that BY affects the volume of bile and the relative amounts of dihydroxy- and trihydroxycholanic acids. The significance of the latter observation will be discussed in terms of the biosynthesis of bile acids from cholesterol.

### MATERIAL AND METHODS

### *Material*

Stock male rats of the G.G. strain (Gilbert, Gillman, Loustalot and Lutz, 1958) were reared on a full basal diet (*ibid.*) to which BY (0·06 per cent of diet) was added as a 3 per cent solution in arachis oil, from the age of 3–4 months (these rats are henceforth referred to as "BY rats"). This regimen produces liver tumours in most rats of our stock after 6–8 months. At the same time control rats of the same age and sex were reared on the basal diet alone.

### *Collection of bile*

When BY had been fed for periods varying from 2 to 5 months, the bile ducts of groups of 3–5 BY rats weighing 200–300 g. were fistularized and the bile was collected in glass saddles (van Zyl, 1957). The saddles allow the rats to move around freely in contrast to the restraining cages usually employed. With each group of BY rats a corresponding number of control rats was also fistularized.

After the operation the majority of the rats secreted bile copiously for several days at least. They were maintained on the same diet except that 0·85 per cent NaCl was supplied instead of water. A preparation containing 10,000 i.u. penicillin and 50 mg. streptomycin was injected subcutaneously after operation and on the subsequent two days.

Preliminary experiments showed that the presence of thymol did not interfere with the bile acid analyses. Accordingly 0·2 ml. of 5 per cent thymol in ethanol was added every evening as preservative to each saddle. The bile was collected from the saddles twice daily and where necessary was stored at $-15^\circ$ C. For most of the experiments the bile from each rat was pooled from the second to the fourth days after cannulation. Where the bile from all three days was not available due to spillage, blockage of the cannula or other accidents, the bile was pooled from as many of these days as possible. Bile secreted on the day before death was discarded. Some rats died after a varying period of bile drainage and some were killed with ether, as detailed later. After death the rats were examined and the livers were preserved in formalin. Sections for histological examination were taken from at least the left lateral, left median and right median lobes.

### *Analysis of the bile acids*

The bile acids were extracted by a modification of the method of Mosbach, Kalinsky, Halpern and Kendall (1954) from three aliquots of each bile sample, each aliquot consisting of $\frac{1}{3}$–$\frac{1}{2}$ of the mean daily production of bile, corresponding to 4–8 ml. bile and 5–20 mg. bile acids. Each aliquot was heated with NaOH (final concentration 1·5 N) in a sealed pyrex test-tube at 120° for 4 hours to hydrolyze the conjugated bile acids. The hydrolysate was extracted with petrol ether (40–60° ; 2 × 20 ml.), which was then re-extracted with water (2 × 10 ml.). The combined aqueous phase was acidified with HCl and extracted with re-distilled ether (5 × 20 ml.). The ether extract was shaken with water (5 × 5 ml.) to remove the HCl, dried over sodium sulphate, evaporated and stored over $P_2O_5$ in a dessicator. Storage for several months did not affect the subsequent analyses.

The trihydroxycholanic acids (THA) and dihydroxycholanic acids (DHA) in the samples were analysed by quantitative, reversed phase, partition chromatography on columns of non-wetting kieselguhr. The method employed was an extension of the qualitative method of Bergström and Sjövall (1951) and Sjövall (1953) and has been briefly described in previous publications (Mirvish, 1957, 1958). A 1 ml. siphon, a mixing vessel and 0·01 N methanolic NaOH in a burette were used to titrate each 1 ml. fraction as soon as it was eluted. Each titration was carried out to an end-point of pH 8·8 using a Beckman Model G pH meter, as recommended by Dr. A. Antonis (private communication). The meter was connected by shielded wire to a glass electrode and a calomel half-cell in which was inserted a bridge of agar-saturated KCl. The glass electrode and agar bridge dipped into the mixing vessel through side arms sealed with rubber tubing. The nitrogen was passed through soda lime before entering the mixing vessel.

The solvents for chromatograpy were freshly mixed each day and a trace of HCl (0·0002 N) was added to the moving phase to prevent losses on the column. The acids were applied as a solution in moving phase (5 ml.) immediately after 50 ml. of moving phase had been run through the column. The flow rate was adjusted to 1 ml. per 90–100 seconds. A " base line " figure of 0·015–0·030 ml., which corresponded to the added HCl and was best determined after elution of all the bile acids, was subtracted from the results of each titration. Controls were run at frequent intervals with mixtures of pure cholic and deoxycholic acids, by simple chromatography and by chromatography following alkali treatment and extraction.

Two of the three aliquots from each bile sample were chromatographed and, if the results differed by more than 2·0 mg. for THA (total 10–20 mg.) and 0·5 mg. for DHA (total 0–4 mg.), then the third aliquot was also chromatographed. The results for THA and DHA were expressed as concentrations or as mg./24 hours/100 g. body weight, as calculated from the mean titration figure, the bile volume and the mean weight of the rat on the days during which the bile was secreted. The bile volume was expressed similarly as ml./24 hours/100 g. body weight.

*Accuracy of the analytical method*

Mixtures of 10 mg. cholic acid and 2 mg. deoxycholic acid were eluted from the columns with recoveries of 95 ± 3 per cent and 119 ± 10 per cent respectively (A ± B signifies mean and standard deviation throughout this paper). When similar amounts of the acids were added to bile samples before alkali treatment, the acids were recovered in yields of 80–110 per cent (Mirvish, 1958).

The accuracy of the complete extraction and estimation was tested by analysis of the difference between the duplicate values obtained in the present investigation. The standard deviation ($S$) was calculated for the error of the average of two determinations on the same sample using the formula $S^2 = \Sigma(x_1 - x_2)^2/4n$, where $x_1$ and $x_2$ are the two values and $n$ is the number of samples. The results were 1·0 mg. for THA (control and BY rats), 0·15 mg. for DHA (controls) and 0·27 mg. for DHA (BY rats). These values should be compared with the average figures for all results (11·6, 1·39 and 1·20 mg. respectively—see Table I) and are in the same units, namely mg./24 hours/100 g. body weight.

### RESULTS

### *Time of Survival and Loss of Weight after Cannulation*

Six control rats died within the first six days after cannulation, nine died on days 7–9, and the remaining 24 controls were killed with ether ; 13 on days 5–10, nine on days 11–13 and two on days 19–20. In contrast, 16 of the 34 BY rats died within the first six days after cannulation, eight died on days 7–10 and three on days 16–20. In addition, seven BY rats were killed with ether on days 5–6. It is clear from these results that feeding BY shortens the time of survival after cannulation of the bile duct.

The BY rats lost more weight between cannulation and death than did the controls. For example, the four BY rats which survived the operation for 10 days or more each lost 92–121 g. after cannulation, whereas no control rat lost more than 60 g. The BY rats lost weight steadily at a rate of 4–8 g./day throughout the period of bile drainage ; the controls lost weight at a similar rate until the fourth day, but thereafter the rate fell to 1·5–3·0 g./day.

### *Changes in Bile Composition Induced by Butter Yellow*

In order to separate effects on bile acid secretion from effects on bile volume, the results are mostly expressed in terms of the secretion per 24 hours per 100 g. body weight, rather than as concentrations.

#### *Daily variations in bile composition*

Analyses were carried out on the bile samples secreted each 24 hours by six control and eight BY rats (Fig. 1). The THA, DHA and bile volume fell in both groups of rats on the second day after cannulation, rose to a maximum on the fourth to sixth day and thereafter decreased at varying rates. The values for THA and bile volume were similar in BY and control groups. On the first day after cannulation the proportion of DHA to THA (Fig. 1*d*) in the BY group was as high as that in the control group, but thereafter the BY figure remained consistently below that for the controls, suggesting that differences in DHA might exist between the two groups of rats after the first day.

The correlation coefficient for the relation between THA and DHA in the daily samples was calculated separately for each rat. In the control group two rats showed no significant correlation, three a 90 per cent and one a 99 per cent correlation. In the BY group two rats showed no correlation, one a 90, one a 95, one a 98, two a 99 and one a 99·9 per cent correlation. The correlations are thus much stronger in the BY group than in the control group, but insufficient data were available to test rigidly the significance of this difference between the two groups of rats.

#### *Bile composition of samples pooled over several days*

In view of the results of the daily analyses, in subsequent experiments the bile secreted by each rat during the second, third and fourth days after cannulation was pooled before investigation, in order to reduce the number of rather tedious analyses. Thirty-four BY and 39 control rats were examined in this way, including eight BY and six control rats where the mean bile composition for the three days was calculated from the daily analyses. The values obtained for THA, DHA ad bile volumes are summarized in Table I. The relationship between THA and DHA is shown for each rat in Fig. 2 and 3. For reasons which will become apparent

FIG. 1.—Daily variations in bile composition. Trihydroxyacids (THA), dihydroxyacids (DHA) and bile volume are expressed in mg. or ml./24 hours/100 g. body weight. The curves represent average figures for six control and for eight butter yellow rats.

below, the results in Table I are shown not only for all BY and all control rats, but also separately for those rats of each group with a THA exceeding 10 mg./day/100 g. and for those with less than 10 mg. THA.

Three main conclusions can be drawn from Table I.

Firstly, though there was a wide scatter of THA from rat to rat, there was no significant difference in THA distribution between BY and control rats. As THA

is by far the major fraction of the bile acids, it is concluded that BY did not significantly affect the total secretion of bile acids.

Secondly, BY affected the production of DHA under certain defined conditions. If all the results are taken into account irrespective of the amount of THA secreted, the mean DHA is not significantly different in the BY and control groups, though

FIG. 2.—Relationship between trihydroxyacids (THA (and dihydroxyacids (DHA) in the control series, for bile pooled on the second to fourth days after cannulation. THA and DHA are expressed in mg./24 hours/100 g. body weight. AA = regression line (DHA = 0·176 × THA − 0·65). The nine points below BB constitute the " low DHA group ". Points for rats with *any* degree of bile duct hyperplasia and/or necrosis are differentiated from points for rats with relatively normal livers.

TABLE I.—*Trihydroxyacids (THA), Dihydroxyacids (DHA) and Bile Volume in Control Rats and Rats Fed Butter Yellow, for Bile Pooled on the Second to Fourth Days After Cannulation*

| | Total | | THA less than 10 mg. | | THA more than 10 mg. | |
|---|---|---|---|---|---|---|
| | Control | BY | Control | BY | Control | BY |
| Number of rats | 39 | 34 | 16 | 10 | 23 | 24 |
| THA (mg.) | 11·6±4·75 | 12·4±4·04 | 7·2±1·65 | 7·4±1·63 | 14·7±3·56 | 14·5±2·58 |
| DHA (mg.) | 1·39±0·99 | 1·20±0·61 | 0·58±0·38 | 0·57±0·47 | 1·96±0·90 | 1·47±0·44 |
| Bile volume (ml.) | 5·2±1·40 | 6·20±1·58 | 4·5±1·02 | 5·1±1·45 | 5·7±1·46 | 6·7±1·37 |

the variability of DHA is definitely reduced in the BY rats (98 per cent level). However, if only results from rats secreting more than 10 mg. THA are considered, then the mean value for DHA is significantly lower (95 per cent level) in the BY group. No BY rat secreted more than 2·2 mg. DHA, though the DHA of seven of the 39 controls exceeded this value. BY thus caused a reduction in DHA, but only when a comparatively large amount of THA was produced.

Thirdly, the daily volume of bile was greater in the BY rats than in the normals when all rats were considered irrespective of the value for THA (99 per cent level

FIG. 3.—Relationship between trihydroxyacids (THA) and dihydroxyacids (DHA) in the butter yellow series, for bile pooled on the second to fourth days after cannulation. THA and DHA are expressed in mg./24 hours/100 g. body weight. AA = regression line (DHA = 0·122 × THA − 0·32). The rats are graded according to the position of their points relative to the lines, as indicated by the symbols 2+ to 2− on the right of the figure. Points for rats with *severe* bile duct hyperplasia (3+ grading) and/or *severe* liver necrosis (2+ grading) are distinguished from points for rats with milder liver damage.

of significance), and when only those rats were considered with more than 10 mg. THA (95 per cent level). No significant differences in volume were found for rats with less than 10 mg. THA.

BY feeding thus led to a decrease in the secretion of DHA for the group with more than 10 mg. THA, and to an increase in bile volume. BY should therefore also produce a decrease in the *concentration* of DHA (daily amount of DHA divided by daily volume). No significant decrease in the concentration of DHA is noted if all results are considered. If only those rats secreting more than 10 mg. THA/day/100 g. are taken into account, the concentration of DHA is 38·1 ± 22·3 mg./100 ml. bile in the controls and 22·5 ± 7·94 mg./100 ml. in the BY group

(significantly different at the 99 per cent level); the corresponding concentrations of THA are 276 ± 90 and 223 ± 98 mg./100 ml. (no significant difference).

Both BY and control groups showed significant correlations between THA and DHA (99·9 per cent level) and between THA and volume (99 per cent level). In addition, the BY group showed a striking correlation (99·9 per cent level) between DHA and bile volume, though the control group here showed no correlation at all.

The first order regression lines were calculated for DHA as a function of THA in the BY and control groups (Fig. 2 and 3) and for DHA as a function of volume in the BY group. The regression lines for the DHA/THA diagrams do not pass through the origin, so that little or no DHA is produced when THA falls below 3–4 mg. As the amount of THA rises above this figure, the proportion of DHA to THA increases rapidly, especially in the control series. The control rats with a very high THA seem to show a DHA even greater than that expected from the calculated regression line, suggesting that the true regression line may here be of the second order. These observations demonstrate the importance of determining total daily secretions and not merely the ratios between the different bile acids.

The changes in bile composition induced by BY may be summarized as follows: (1) DHA (expressed either as daily secretion or as concentration) was decreased in those cases where the daily secretion of THA exceeded 10 mg./100 g. body weight. This decrease was associated with a decrease in the inter-individual variability of DHA. (2) Bile volume was increased. (3) A strong inter-individual correlation developed between DHA and volume (for the pooled samples from different rats), and (4) the intra-individual correlation between DHA and THA was probably increased (for different daily samples from a single rat). It should be emphasized that BY did not affect the production of THA, and that the DHA/THA ratio rose with an increase in THA, especially in the control series.

*Factors Affecting the Inter-individual Variations in Bile Composition*

An investigation of the factors responsible for the considerable overlap between the results of the BY and control groups might increase the significance of the difference between the two groups of rats. The livers showed variable degrees of acute necrosis and bile duct hyperplasia in both series of rats, so that some of the variations in bile composition might be explained in terms of the extent of liver damage. In the following sections, the interrelationships will be examined between bile composition, liver histology and the reaction of the rat to cannulation.

A. *Relationship between bile composition and liver damage in the control rats*

Eighteen of the 31 livers examined from the control series appeared almost normal, and 13 showed some degree of bile duct hyperplasia and/or necrosis. The extent of each form of liver injury was graded 0, ±, +, 2+ or 3+. A variable degree of bile duct hyperplasia was observed in nine rats (± in four, + in one and 2+ to 3+ in four). A mild necrosis was observed in eight rats (± in five, + in two and 2+ in one). These figures include those from four rats with both types of injury. As rats of the same strain do not normally show spontaneous liver damage, much of the observed injury was probably a consequence of the cannulation and subsequent bile drainage.

Three of the rats with bile duct hyperplasia had been killed on the fifth day after cannulation, two had died on the seventh day and four had been killed after

8–13 days. Thus in at least some instances bile duct hyperplasia was present at the time when the bile was being collected for analysis, i.e. on the second to fourth day after cannulation, and any association found between bile composition and this form of liver damage is probably direct.

Two of the control rats with necrosis had died on the fifth day after cannulation, three had died or had been killed on days 7–8 and three had been killed on days 11–13. It is thus not clear whether the necrosis developed during the period when the bile was being collected for analysis or later, and the significance of any relationship found between necrosis and bile composition will therefore be obscure.

Nine of the 39 control rats showed points on the DHA/THA graph below line BB (Fig. 2) and were hence deemed to have secreted an unusually low DHA relative to THA. This low DHA group contained seven of the nine rats with bile duct hyperplasia (Fig. 2) ; the liver of one of the remaining members of the low DHA group was normal and the other liver was lost. The mean bile volume was 6·56 ml./day/100 g. body weight for the nine rats with bile duct hyperplasia, compared with 5·20 ml. for all the controls and 6·20 ml. for the BY rats. Bile duct hyperplasia due to cannulation of the bile duct in normal rats apparently affects bile composition in the same way as does BY administration, namely by lowering DHA relative to THA and increasing the bile volume. These changes in bile composition are, therefore, not specific to the process of BY carcinogenesis.

For the 16 control rats which did not show bile duct hyperplasia and which secreted more than 10 mg. THA, the DHA was $2{\cdot}35 \pm 0{\cdot}76$ mg./day/100 g. as compared with $1{\cdot}96 \pm 0{\cdot}90$ mg. for all control rats with more than 10 mg. THA and $1{\cdot}47 \pm 0{\cdot}44$ mg. for the BY rats with more than 10 mg. THA (Table I). Thus exclusion of the control rats with bile duct hyperplasia accentuates the difference in mean DHA between control and BY groups. The difference is now significant at the 99·9 per cent level instead of the 95 per cent level found when all control rats were considered, irrespective of the histology of their livers. Furthermore, the variability in DHA of the control group has been reduced and is now close to that of the BY group.

Cannulation of the bile duct thus led in nine control rats to bile duct hyperplasia. The latter was associated with a bile composition similar to that of the BY rats. These changes in bile composition are therefore not specific. If the results from the control rats with bile duct hyperplasia are excluded, then the difference in mean DHA between BY and control groups is greatly accentuated.

B. *Relationships between bile composition, liver damage and reaction to cannulation in the BY rats*

Two factors, which do not arise in the control series, need to be taken into account when investigating the BY series. Firstly, in addition to cholangiofibrosis and cancer, BY by itself produces bile duct hyperplasia and necrosis of the liver, which closely resemble the similar reactions resulting from cannulation. Secondly, with regard to bile composition it was only necessary in the control series to consider the correlation between DHA and THA ; in the BY series the correlation between DHA and volume, which is absent in the controls, also has to be taken into account.

In view of these complications, a system was adopted whereby the performance of each BY rat was graded with respect to each of four factors : (1) the relation of DHA to THA, (2) the relation of DHA to bile volume, (3) the extent of bile duct hyperplasia and (4) the extent of liver necrosis.

A grading was assigned to each rat according to the position of its point on the DHA/THA graph relative to the regression line, positive gradings signifying a relatively high DHA and negative gradings a low DHA (Fig. 3). A second grading was assigned similarly from a graph of DHA plotted against bile volume. The two gradings were of opposite sign for four rats ; three of these produced less than 8·5 mg. THA and the fourth produced 10·0 mg. THA. For eight rats one or both of the points lay very close to the regression line (i.e. with grading $\pm$). For the remaining 22 rats the two gradings were both definitely positive or both definitely negative. Thus DHA tended either to be high relative to both THA and bile volume, or to be low relative to both these factors, except where the total production of bile acids was very low.

In order to avoid dealing with gradings for both relationships (DHA/THA and DHA/Volume), the two gradings were added to give a composite " bile grading ", except in the four cases with gradings of opposite sign, where no " bile grading " was assigned. This composite grading will be used in the ensuing discussion as a general index of the extent to which bile composition departs from the normal. A high bile grading (up to 4+) indicates a relatively normal bile composition and a low bile grading (down to 4—) indicates a relatively abnormal bile composition.

Necrosis was present in 27 of the 33 livers examined and bile hyperplasia in 22. Gradings from 0 to 3+ were assigned for each type of liver damage, as with the control rats. Five livers showed cholangiofibrosis and/or small cancerous foci.

We may now attempt to determine whether variations in the bile composition of BY rats are associated with variations in the reaction of the liver to cannulation, as in the case of the control rats. In order to facilitate analysis, the data were grouped according to the values for bile grading, days lived after cannulation and length of time for which BY was fed (Tables II, III and IV). The seven cases with less than 8·5 mg. THA, together with the rat which secreted 10·0 mg. THA but showed gradings for DHA/THA and for DHA/Volume of opposite sign, are listed as a separate " low bile acid group " in Table II and are omitted from consideration in Tables III and IV.

The bile gradings for the five rats with cancerous nodules and/or cholangiofibrosis varied widely, so that neither cancer nor cholangiofibrosis appears to affect bile composition, apart from the general effects of BY.

The eight rats of the low bile acid group showed definite liver necrosis (with one exception) and a variable degree of bile duct hyperplasia (Table II). All but

TABLE II.—*Extent of Liver Damage in the Butter Yellow Rats, Grouped According to Bile Grading*

| Bile grading | Number of rats | Degree of necrosis | | | | Degree of bile duct hyperplasia | | | | |
|---|---|---|---|---|---|---|---|---|---|---|
| | | 2+ | 1+ | ± | 0 | 3+ | 2+ | 1+ | ± | 0 |
| 3+ to 4+ | 9 | 5 | 3 | 1 | — | 3 | 1 | 2 | 1 | 2 |
| ± to 2+ | 7 | 2 | 2 | 2 | 1 | 3 | 2 | 1 | — | 1 |
| 1— to 4— | 10 | — | 3 | 3 | 4 | 1 | — | 3 | 2 | 4 |
| *Low bile acid | 8 | 2 | 3 | 1 | 1 | 2 | — | 1 | 1 | 3 |
| All values | 34 | 9 | 11 | 7 | 6 | 9 | 3 | 7 | 4 | 10 |

* One liver was lost here.

one of these rats died within six days of the cannulation. Total or partial obstruction of the bile flow leads within 48 hours to liver necrosis and bile duct hyperplasia (Cameron and Oakley, 1932 ; Gillman, Gilbert and Spence, 1954), so that biliary obstruction was probably the main cause in this group of the low production of bile acids, the necrosis and the early death. These cases are not considered in the following two paragraphs.

Nineteen rats were not deliberately killed and did not belong to the low bile acid group. Of these, all ten rats living for seven or more days after cannulation showed positive bile gradings, including seven out of the nine rats with bile gradings of 3+ or 4+ (Table III). The five rats with negative bile gradings lived for

TABLE III.—*Bile Gradings and Extent of Liver Damage in the Butter Yellow Rats, Grouped According to Manner of Death and Days Lived After Cannulation*

| Manner of death | Time of survival after operation (days) | Number of rats | Bile grading 3+ to 4+ | ± to 2+ | 1− to 2− | 3− to 4− | Necrosis 2+ | 1+ | ± | 0 | Bile duct hyperplasia 3+ | 2+ | 1+ | ± | 0 |
|---|---|---|---|---|---|---|---|---|---|---|---|---|---|---|---|
| Died . . | 3–6 | 9 | 2 | 2 | 5 | — | 1 | 4 | 2 | 2 | 1 | — | 4 | — | 4 |
| Died . . | 7–20 | 10 | 7 | 3 | — | — | 6 | 1 | 2 | 1 | 5 | 1 | 2 | 1 | 1 |
| Killed with ether | 5–6 | 7 | — | 2 | 1 | 4 | — | 3 | 2 | 2 | 1 | 2 | — | 2 | 2 |

less than six days. Cannulation thus precipitates death least rapidly in those rats with the most positive bile gradings, i.e. in those rats with bile compositions which approach closest to the normal.

The maximum liver damage was found in rats with high bile gradings, i.e. with relatively high values for DHA (Table II, Fig. 3), in contrast with the exactly opposite position found for the control rats (Fig. 2). The BY rats thus showed a paradoxical association of liver damage with a relatively normal bile composition. The explanation of the paradox is clearly that the BY rats with high bile gradings survived the longest after cannulation (preceding paragraph) and so were more likely to develop liver injury. Thus 2+ necrosis and/or 3+ bile duct hyperplasia were found in seven of the ten rats living for seven or more days after cannulation, but in only three of the 16 rats which died or were killed before the seventh day (Table III). The extent of liver injury was much greater than that expected in non-cannulated BY rats, and it is clear that most of the injury observed in the BY rats developed as a response to prolonged bile drainage. In the control rats liver damage was far less severe, even in those rate surviving for a considerable period after cannulation. BY thus increased the vulnerability of the liver to prolonged bile drainage. This increase in vulnerability was possibly responsible for the BY rats dying sooner after cannulation than the controls.

A correlation has clearly not been established in the BY rats between bile composition and the condition of the liver *at the time when the bile was being collected for analysis*, i.e. on the second to fourth days after cannulation. Bile composition is less variable in the BY rats then in the controls, and so is apparently not as easily affected by differing responses to cannulation.

The extent of liver necrosis and of bile duct hyperplasia tended to increase as the period of BY feeding was lengthened from 63 to 153 days (Table IV). The mean bile gradings, however, did not alter significantly during this period (Table IV). The reduction in DHA from the normal values had thus occurred to the

TABLE IV.—*Bile Gradings and Extent of Liver Damage in the Butter Yellow Rats, Grouped According to the Length of Time for which Butter Yellow was Fed*

| Days on butter yellow | Number of rats | Bile grading | | | | Necrosis | | | | Bile duct hyperplasia | | | | |
|---|---|---|---|---|---|---|---|---|---|---|---|---|---|---|
| | | 3+ to 4+ | ± to 2+ | 1− to 2− | 3− to 4− | 2+ | 1+ | ± | 0 | 3+ | 2+ | 1+ | ± | 0 |
| 63– 81 . | 6 | 2 | 1 | 3 | — | 1 | — | 3 | 2 | — | — | 3 | — | 3 |
| 98–118 . | 12 | 3 | 7 | 1 | 1 | 4 | 4 | 2 | 2 | 6 | 2 | 1 | 1 | 2 |
| 138–153 . | 8 | 4 | — | 1 | 3 | 2 | 4 | 1 | 1 | 1 | 1 | 2 | 2 | 2 |

same extent after 63–81 as after 138–153 days of BY feeding, and probably took place within the first 60 days.

From the foregoing it would appear that : (1) Cases with a high DHA relative to THA also show a high DHA relative to bile volume. (2) The presence of cancerous nodules or cholangiofibrosis does not affect bile composition, apart from the general effects of BY. (3) Eight rats show a low secretion of total bile acids, associated with extensive necrosis and early death after cannulation. (4) Cannulation leads to death more rapidly in those BY rats having the more abnormal bile compositions. (5) BY rats are vulnerable to the effect of bile drainage and usually develop extensive liver damage about seven days after cannulation. (6) Bile composition is probably affected within 60 days of BY feeding.

### DISCUSSION

The findings will now be discussed with special reference to the effects of cannulating the bile duct, cholesterol metabolism, and conditions other than BY treatment which are known to affect bile acid metabolism. A mechanism involving changes in the rate of 12-hydroxylation will be proposed to explain observed changes in DHA/THA ratios, and the possible significance will be examined of hydroxylation in general for the understanding of cancer.

Death follows cannulation of the bile duct more rapidly in BY rats than in the controls, and more rapidly in BY rats with low bile gradings than in BY rats with high bile gradings. The rats could have died from a deficiency of a fat-soluble vitamin, e.g. vitamin K (some rats bled very easily after a few days of bile drainage and a deficiency of vitamin K is well known to result after the bile duct has been fistularized in man and in the dog).

The daily production of bile acids in the intact rat is only about 2 mg./100 g. body weight (Linstedt and Samuelsson, 1959) as compared with the 10–20 mg./100 g. found for cannulated rats in the present and previous investigations (Eriksson, 1957). Thus bile acid production is greatly increased by cannulation of the bile duct. In a rat with an intact entero-hepatic circulation the bile acids returning in the portal vein probably inhibit the further production of bile acids by the liver (Bergström and Danielsson, 1958). Now the bile of BY rats secreted on the first day after cannulation appears to show a normal DHA/THA ratio (Fig. 1*d*). As this bile largely represents bile that had been circulating in the intact rat, it seems likely that BY does not reduce DHA in the intact rat. BY has thus been demonstrated to lower DHA only when the production of bile acids is strained to a maximum due to the cannulation (and even then the effect is only noted when bile acid production rises above 10 mg./100 g.).

BY did not affect the total production of bile acids in the cannulated rat. This result does not support the view that the raised serum cholesterol which is apparently associated with bile duct hyperplasia (Gillman, Gilbert and Spence, 1954), is due to a decreased degradation of cholesterol to the bile acids.

Eriksson (1957) showed that the DHA (actually chenodeoxycholic acid was measured) rose from the normal 10–20 per cent of the total bile acids to 50–60 per cent on feeding desiccated thyroid and fell to 5 per cent on feeding thiouracil. The action of BY on DHA could therefore be due to a depression of thyroid function. The latter is not known to be affected by BY, though thiouracil delays the production of hepatomas by BY (Paschkis, Cantarow and Stasney, 1948; Harris and Clowes, 1952).

Carey (1958), Rudman and Kendall (1957) and Osborn, Wootton, da Silva and Sherlock (1959) have recently reported on the levels of serum bile acids in various types of jaundice. Though the results from the three groups of workers differ considerably, it is agreed that the THA/DHA ratio is almost always below 1·0 in cases of portal cirrhosis, and is above this figure in obstructive jaundice. Carey found that six of the seven cases of obstructive jaundice due to carcinoma (of various types) showed THA/DHA ratios greater than 3·8, compared with two of the ten patients with other types of obstructive jaundice. Thus DHA production may be relatively depressed in human cancer, reminiscent of the position in the BY rats. The cancer cases examined by Osborn *et al.* showed no depression of DHA.

If one may speculate, the level of DHA relative to that of THA could serve as a useful indicator of liver metabolism in various diseases, especially if cirrhosis on the one hand and bile duct hyperplasia and liver cancer on the other hand are found to affect the proportion of DHA in opposite directions. It is possible that conditions only become favourable for the development of liver cancer when DHA secretion is diminished. The bile acids may themselves influence liver structure and function on their return to this organ via the portal vein; the bile acids returning to the liver are already known to regulate the further synthesis of bile acids. Finally, it should not be forgotten that changes in bile acid composition could also affect the digestion and absorption of lipids.

*Possible mechanism for changes in the relative amounts of DHA and THA*

Cholic acid (3 : 7 : 12-trihydroxycholanic acid) is the main acid of the THA fraction, apart from minor amounts of 3 : 6 : 7-trihydroxycholanic acids (Hsia, Matschiner, Mahowald, Elliott, Doisy, Thayer and Doisy, 1957, 1958). Chenodeoxycholic acid (3 : 7-dihydroxycholanic acid) is the main acid of the DHA fraction (Bergström and Sjövall, 1954). The depressed DHA in the BY rats is thus due, presumably, to changes in the relative rates of synthesis of cholic and chenodeoxycholic acids. According to Bergström (1959), these syntheses proceed as follows: The first step for both acids is the hydroxylation of cholesterol to form $7\alpha$-hydroxycholesterol. In the case of chenodeoxycholic acid the 3-hydroxy group is then converted from the $\beta$ to the $\alpha$ configuration, the double bond at $C_5$–$C_6$ is saturated and finally the side chain is oxidized with the removal of three carbon atoms. The identical changes take place during the formation of cholic acid, except that a $12\alpha$-hydroxy group is inserted at some stage before side-chain oxidation. Chenodeoxycholic acid is not directly converted into cholic acid.

It appears likely that the relative amounts of DHA and THA are controlled, in part at least, by the rate of 12-hydroxylation, as this reaction constitutes the only difference between the syntheses of cholic and chenodeoxycholic acids. In the present paper the production of DHA relative to that of THA has been modified in three circumstances : (1) Feeding BY reduces the DHA (at high levels of THA). (2) The proportion of DHA rises as the total production of bile acids rises, especially in the control series. (3) In BY rats the amount of DHA rises with an increase in bile volume.

If the effects are due in all three circumstances to modifications in the rate of 12-hydroxylation, it follows that : (1) feeding BY leads to an acceleration of 12-hydroxylation, (2) as more bile acids are formed, the enzymes controlling 12-hydroxylation can cope with a smaller proportion of the bile acids, so that more DHA is formed, and (3) in BY rats secreting a large volume of bile, the proportion of bile acids which escapes 12-hydroxylation is increased, possibly because the bile is swept out of the liver lobule before hydroxylation can be completed. For unknown reasons the last factor does not operate in normal rats. It has also been mentioned that there is an increased formation of DHA in hyperthyroidism and in liver cirrhosis. This increase could also be due to a decrease in the rate of 12-hydroxylation, as already proposed by Carey (1958) in the case of cirrhosis.

It will be recalled that on cannulation of the bile duct the liver is stimulated to produce an abnormally large amount of bile acids. This load which has been placed on the liver by cannulation has, according to the above hypothesis, disclosed a slight defect in the capacity of the liver to hydroxylate bile acids, which might otherwise have escaped detection.

*The relation between cancer and hydroxylations in the liver*

An increased rate of hydroxylation in the liver may not be confined to the possible acceleration of 12-hydroxylation by BY. Thus the intraperitoneal injection of benzpyrene, methylcholanthrene and other polycyclic hydrocarbons (carcinogenic and non-carcinogenic) leads to an increase in liver benzpyrene hydroxylase (Conney, Miller and Miller, 1957). Administration of the carcinogen acetylaminofluorene leads to cancer of the urinary bladder if tryptophan is also fed (Dunning, Curtis and Maun, 1950). Now 3-hydroxyanthranilic acid and 3-hydroxykynurenine are compounds which are carcinogenic to the bladder (Allen, Boyland, Dukes, Horning and Watson, 1957) and are normally formed from tryptophan in the liver by a series of reactions including a hydroxylation, and it seems that acetylaminofluorene might act by accelerating the formation of these two compounds by the liver.

Another connection between liver hydroxylation and cancer is that the liver can hydroxylate several aromatic amines in the ortho position to form very active carcinogens, as proposed by Clayson (1953). The hydroxylations involved in the conversions of tryptophan discussed above are of this type. Amines such as $\beta$-naphthylamine are similarly converted into carcinogenic *o*-hydroxy derivatives (Boyland, 1958). Acetylaminofluorene is converted by the liver into *o*-hydroxy derivatives which may be the active agents responsible for cancer of the liver due to this carcinogen (Weisburger, Weisburger and Morrix, 1957).

Cancer and hydroxylations may thus be linked in two ways: (1) Following Clayson's hypothesis, the liver hydroxylates amines to form carcinogenic *o*-

hydroxyamines, and (2) the rate of certain hydroxylations in the liver may be increased during the stage of induction of cancer.

### SUMMARY

Details are described for a method of estimating the trihydroxy-acids (THA) and dihydroxyacids (DHA) in rat bile, using reversed phase partition chromatography. The method was applied to the analysis of samples of rat bile, collected on the second to fourth days after cannulation of the bile duct from 39 control rats and 34 rats fed butter yellow (BY) for 63–153 days.

After cannulation the BY rats died sooner and lost weight faster than the controls. The cannulation and subsequent bile drainage gave rise in many rats of both series to bile duct hyperplasia and necrosis of the liver. The BY rats were very vulnerable to the effects of bile drainage and usually developed extensive liver damage about seven days after cannulation.

Although the secretion of THA was similar in both groups of rats, the BY rats showed a decrease in DHA for those rats with a daily secretion of THA exceeding 10 mg./100 g. body weight. The BY rats also showed an increase in bile volume, and a strong correlation between DHA and bile volume which was entirely absent in the controls. The proportion of DHA to THA rose as THA secretion was increased, especially in the control series. In the control series the nine rats with bile duct hyperplasia showed changes in bile composition characteristic of those induced by BY. Exclusion of the results from these rats greatly accentuated the difference in DHA between control and BY rats. In the BY series cannulation was followed by death quickest in those rats with the most abnormal bile composition.

The significance of the depression in DHA by BY feeding is discussed in terms of other conditions which affect bile acid ratios. A mechanism involving alterations in the rate of 12-hydroxylation is proposed to explain the effect of BY feeding on DHA, the increase in the proportion of DHA with a rise in THA, and the correlation in the BY rats between DHA and bile volume. The possible significance is examined of hydroxylations in general for the understanding of cancer.

Thanks are expressed to N. G. N. Matthews and J. Makunga for excellent technical assistance and to Miss J. van Veen for carrying out the cannulations. Mr. W. Lutz very kindly carried out the statistical analysis of the data. The study was greatly facilitated by a grant made to one of us (S. M.) by the National Cancer Association of South Africa, to which body grateful acknowledgment is made.

### REFERENCES

Allen, M. J., Boyland, E., Dukes, C. E., Horning, E. S. and Watson, J. G.—(1957) *Brit. J. Cancer*, **11**, 212.

Bergström, S.—(1959) ‘ The Biosynthesis of Terpenes and Sterols’. A Ciba Foundation Symposium. London (J. A. Churchill & Co.) p. 185.

*Idem* and Danielsson, H.—(1958) *Acta physiol. scand.*, **43**, 1.

*Idem* and Sjövall, J.—(1951) *Acta chem. scand.*, **5**, 1267.—(1954) *Ibid.*, **8**, 611.

Berman, C.—(1958) *Advanc. Cancer Res.*, **5**, 56.

Booth, J. and Boyland, E.—(1957) *Biochem. J.*, **66**, 73.

Boyland, E.—(1958) *Brit. med. Bull.*, **14**, 153.

CAMERON, G. R. AND OAKLEY, C. L.—(1932) *J. Path. Bact.*, **35**, 769.
CAREY, J. B.—(1958) *J. clin. Invest.*, **37**, 1494.
CLAYSON, D. B.—(1953) *Brit. J. Cancer*, **7**, 460.
CONNEY, A. H., MILLER, E. C. AND MILLER, J. A.—(1957) *J. biol. Chem.*, **228**, 753.
COOK, J. W., KENNAWAY, E. L. AND KENNAWAY, N. M.—(1940) *Nature, Lond.*, **145**, 627.
DUNNING, W. F., CURTIS, M. R. AND MAUN, M. E.—(1950) *Cancer Res.*, **10**, 454.
ERIKSSON, S.—(1957) *Proc. Soc. exp. Biol. N.Y.*, **94**, 578, 582.
FIESER, L. F., GREENE, T. W., BISCHOFF, F., LOPEZ, G. AND RUPP, J. J.—(1955) *J. Amer. chem. Soc.*, **77**, 3928.
GILBERT, C., GILLMAN, J., LOUSTALOT, P. AND LUTZ, W.—(1958) *Brit. J. Cancer*, **12**, 565.
GILLMAN, J., GILBERT, C. AND SPENCE, L.—(1954) *Cancer*, **7**, 1109.
GREENSTEIN, J. P.—(1954) ' The Biochemistry of Cancer '. New York (Academic Press).
HAROLD, F. M., FELTZ, J. M. AND CHAIKOFF, I. L.—(1955) *Amer. J. Physiol.*, **183**, 459.
HARRIS, P. N. AND CLOWES, G. H. A.—(1952) *Cancer Res.*, **12**, 471.
HSIA, S. L., MATSCHINER, J. T., MAHOWALD, T. A., ELLIOTT, W. A., DOISY, E. A., Jr., THAYER, S. A. AND DOISY, E. A.—(1957) *J. biol. Chem.*, **225**, 811 ; **226**, 667.—(1958) *Ibid.*, **230**, 573.
LINSTEDT, S. AND SAMUELSSON, B.—(1959) *Ibid.*, **234**, 2026.
MIRVISH, S.—(1957) *S.Afr. J. med. Sci.*, **22**, 158.—(1958) *Ibid.*, **23**, 33.
MOSBACH, E. S., KALINSKY, H. J., HALPERN, E. AND KENDALL, F. E.—(1954) *Arch. Biochem. Biophys.*, **51**, 402.
OSBORN, E. C., WOOTTON, I. D. P., DA SILVA, L. C. AND SHERLOCK, S.—(1959) *Lancet*, ii, 1049.
PASCHKIS, K. E., CANTAROW, A. AND STASNEY, J.—(1948) *Cancer Res.*, **8**, 257.
RUDMAN, D. AND KENDALL, F. E.—(1957) *J. clin. Invest.*, **36**, 530.
SIPERSTEIN, M. D., JAYKO, M. E., CHAIKOFF, I. L. AND DAUBEN, W. G.—(1952) *Proc. Soc. exp. Biol. N.Y.*, **81**, 720.
SJÖVALL, J.—(1953) *Acta physiol. scand.*, **29**, 232.
SPAIN, J. D. AND GRIFFIN, A. C.—(1957) *J. nat. Cancer Inst.*, **18**, 693.
VAN ZYL, A.—(1957) *J. Endocrin.*, **16**, 213.
WEISBURGER, J. H., WEISBURGER, E. K. AND MORRIS, H. P.—(1957) *Science*, **125**, 503.